# Evaluation of an educational program for essential newborn care in resource-limited settings: Essential Care for Every Baby

**DOI:** 10.1186/s12887-015-0382-z

**Published:** 2015-06-24

**Authors:** Anu Thukral, Jocelyn Lockyer, Sherri L. Bucher, Sara Berkelhamer, Carl Bose, Ashok Deorari, Fabian Esamai, Sonia Faremo, William J. Keenan, Douglas McMillan, Susan Niermeyer, Nalini Singhal

**Affiliations:** All India Institute of Medical Sciences, New Delhi, India; University of Calgary, Calgary, Canada; Indianappolis Indianna University School of Medicine, ., USA; University at Buffalo, SUNY, Buffalo, NY USA; University of North Carolina, Chapel Hill, USA; Moi University School of Medicine, Eldoret, Kenya; St. Louis University, St Louis, USA; Dalhousie University, Halifax, Canada; University of Colorado, Aurora, USA

## Abstract

**Background:**

Essential Care for Every Baby (ECEB) is an evidence-based educational program designed to increase cognitive knowledge and develop skills of health care professionals in essential newborn care in low-resource areas. The course focuses on the immediate care of the newborn after birth and during the first day or until discharge from the health facility. This study assessed the overall design of the course; the ability of facilitators to teach the course; and the knowledge and skills acquired by the learners.

**Methods:**

Testing occurred at 2 global sites**.** Data from a facilitator evaluation survey, a learner satisfaction survey, a multiple choice question (MCQ) examination, performance on two objective structured clinical evaluations (OSCE), and pre- and post-course confidence assessments were analyzed using descriptive statistics. Pre-post course differences were examined. Comments on the evaluation form and post-course group discussions were analyzed to identify potential program improvements.

**Results:**

Using ECEB course material, master trainers taught 12 facilitators in India and 11 in Kenya who subsequently taught 62 providers of newborn care in India and 64 in Kenya. Facilitators and learners were satisfied with their ability to teach and learn from the program. Confidence (3.5 to 5) and MCQ scores (India: pre 19.4, post 24.8; Kenya: pre 20.8, post 25.0) improved (*p* < 0.001). Most participants demonstrated satisfactory skills on the OSCEs. Qualitative data suggested the course was effective, but also identified areas for course improvement. These included additional time for hands-on practice, including practice in a clinical setting, the addition of video learning aids and the adaptation of content to conform to locally recommended practices.

**Conclusion:**

ECEB program was highly acceptable, demonstrated improved confidence, improved knowledge and developed skills. ECEB may improve newborn care in low resource settings if it is part of an overall implementation plan that addresses local needs and serves to further strengthen health systems.

**Electronic supplementary material:**

The online version of this article (doi:10.1186/s12887-015-0382-z) contains supplementary material, which is available to authorized users.

## Background

Over the past two decades, there has been a significant reduction in under-5 mortality; however, rates of decline of neonatal mortality have been slower, and neonates (<28 days) now account for 44 % of under-5 deaths [1, 2]. Major causes of neonatal deaths include infections, prematurity and asphyxia [3, 4]. Nearly 99 % of all neonatal deaths occur in low and middle-income countries and the majority of these deaths occur during the first 7 days after birth [2]. The main reason for high mortality in resource-limited settings may relate to shortages of competent and adequately skilled health care professionals in community birth facilities [5].

Low-cost interventions, including training in neonatal resuscitation and other elements of basic newborn care, have the potential to reduce deaths from the three main causes of neonatal mortality [6–8]. The World Health Organization (WHO) developed guidelines for essential newborn care that focus on basic interventions, such as resuscitation at birth, early and exclusive breastfeeding, maintenance of normal temperature, hygiene and prevention of infection [9, 10]. A study testing the effectiveness of training birth attendants from rural communities in six countries using a simplified essential newborn care educational program demonstrated that teaching a bundled newborn care curriculum reduces stillbirth [11], and justified the development of a curriculum that could be taught widely. The WHO developed a curriculum to teach essential newborn care that relies on computer technology and is intended to be taught over 4 to 7 days [10]. Essential newborn care has also been taught using other educational methodologies [12–18]. However, the dissemination of the WHO course has been limited perhaps because it is resource intensive and includes extensive use of digital images. WHO ENC course has an extensive clinical workbook and a lengthy guide for facilitators. It has slides requiring technical support. The course in its current format may not be ideal for resource-limited environments. There appeared to be a need for a simple, widely available, skills-based program for training birth attendants in basic newborn care and quality improvement methods to strengthen health systems in resource-limited areas.

In 2010, a private-public consortium led by the American Academy of Pediatrics (AAP) introduced Helping Babies Breathe (HBB), a simplified, low-cost curriculum for teaching newborn resuscitation in resource-limited areas [19–21]. This program incorporates skill-based learning using simulation, peer teaching and learning exercises and a pictorial action plan that guides care. HBB has been shown to be effective in decreasing neonatal mortality and stillbirths [20, 22].

Given the wide acceptance of HBB and in response to requests for a similar course for teaching other elements of essential newborn care, a group of neonatal care specialists in North America, in collaboration with international experts, developed a simplified educational program based on the educational principles used to develop HBB to teach providers the knowledge and skills necessary to deliver essential newborn care. It has a graphical design similar to HBB. The content of this program was based on WHO guidelines for newborn care as outlined in their publication Pregnancy, Childbirth, Postpartum and Newborn Care [9] and the WHO 2010 Essential Newborn Care Course [8]. ECEB curriculum content begins after immediate care at birth and includes care that is delivered during the first day following birth or until the time of discharge from the birth facility. It assumes that resuscitation is taught in a separate program, such as HBB.

The purpose of this study was to test and evaluate the ECEB program in a field setting and modify it as needed. We were specifically interested in learning whether the content could be easily taught and learned, whether facilitators and learners could improve their knowledge and skills, and whether the curriculum (including the program structure, materials and evaluation tools) was appropriately designed.

## Methods

### Program overview

**Fig. 1:**
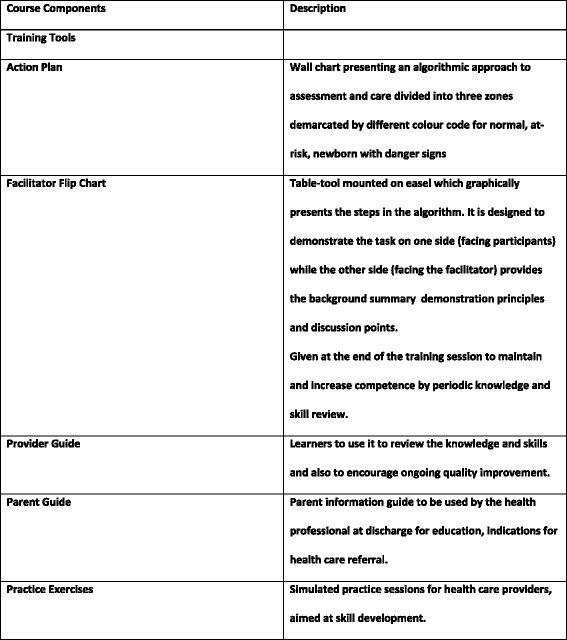
Highlights of Course Design

ECEB is a skills-based educational program intended to be taught over approximately 12 classroom hours. It is designed to be disseminated using a train-the-trainer cascade in which master trainers teach facilitators to deliver the course to providers. Facilitators would typically be experienced clinicians who also have expertise and experience as educators. The target provider audience for the program is facility-based health care workers who deliver newborn care. In this manuscript, they are referred to as learners. The program uses interactive learning strategies with proven effectiveness [23, 24] that include skills practice with a neonatal simulator guided by facilitator demonstration and explanation, small group discussions, and case scenario exercises and role play exercises in which explicit key messages are disseminated. Skills practice and role-play are performed by paired learners; the learners reverse roles and repeat skills practice so that each learner experiences the perspective of both the mother and the provider. Time is devoted to discussion of the potential challenges related to the adoption of recommended practices into the environments in which participants work. Teaching aids include an Action Plan, Facilitator Flip Chart, Provider Guide, and low-cost neonatal simulator. A Parent Guide reinforces key messages in pictorial format. Detailed information about the content of these items is found at http://www.aap.org/en-us/advocacy-and-policy/aap-health-initiatives/global/Pages/eceb.aspx. The highlights of the course design are presented in Fig. 1.

After the initial development of the ECEB program international experts reviewed it through a structured Delphi process. Following this there was a formal consultation with WHO with representation from the 6 regions. WHO experts also provided ongoing input.

### Program evaluation

The program evaluation took place at sites in India and in Kenya. In India, US master trainers (SB, SN, NS) trained facilitators who subsequently trained providers at two sites. The training of providers was observed by the US master trainers at both sites. In Kenya, US master trainers (CB, WK, SLB) trained facilitators who then taught providers in 23 rural health facilities. This training was not observed by master trainers. The evaluation methodology in this study was similar to the evaluations of the HBB [19] and Acute Care of the at Risk Newborn programs [25].

### Evaluation tools and analysis

The following tools and methods were used to evaluate the program:

#### Course assessment survey

To evaluate the ease of using course materials for teaching, the facilitators completed a post-course survey (a) immediately at the end of their training and (b) after they had taught a course. There were 24 items on the post-training survey and 37 items on the second evaluation (after teaching a course). Each item was assigned a numerical score on a scale of 1 to 5 based on ease of use (low to high), and for each item a mean and standard deviation was calculated. Learners also completed a 10-item survey after completion of the course. See Table 1.

**Table 1:** Learners’ post-course evaluation

	India (n = 62)	Kenya (n = 63)
	Mean (SD)	Mean (SD)
1. The course objectives told me what to expect in the course	4.87 (0.38)	4.62 (0.80)
2. I found the course to be well organized	4.87 (0.38)	4.68 (0.78)
3. I had sufficient time to learn the content of the course	4.66 (0.54)	4.48 (0.94)
4. I had sufficient time to practice the skills	4.71 (0.52)	4.49 (0.91)
5. I found the exercises useful	4.92 (0.28)	4.76 (0.76)
6a. Action Plan	4.87 (0.38)	4.69 (0.79)
6b. Facilitator’s Flip Chart	4.90 (0.30)	4.67 (0.79)
6c. “Facilitate practice” exercises	4.85 (0.35)	4.64 (0.80)
6d. Provider Guide	4.95 (0.22)	4.75 (0.77)
6e. Parent Guide	4.89 (0.32)	4.38 (1.02)
7. During the course I had sufficient opportunity to ask questions	4.85 (0.36)	4.67 (0.78)
8. I was able to understand the questions on the multiple choice examination	4.74 (0.44)	4.60 (0.81)
9. I will be able to provide essential newborn care in my work setting	4.84 (0.37)	4.67 (0.82)
10. I was able to understand how to do an OSCE	4.85(0.36)	4.50 (0.86)

#### Confidence survey

Confidence among facilitators and learners was measured using a 15-item questionnaire that was completed prior to and immediately after the course. See Table 2.

**Table 2:** Facilitator & learner pre-post course confidence assessment

	Facilitator				Learner			
	In India (n = 12)		In Kenya (n = 11)		In India (n = 62)		In Kenya (n = 64)	
	Before	After	Before	After	Before Training Mean (SD)	After Training Mean (SD)	Before Training Mean (SD)	After Training Mean (SD)
1. maintain skin**-**to-skin care and when to separate the baby and mother	4.58 (0.67)	5.00 (0)	3.82 (1.47)	5.00 (0)	4.38 (0.86)	5.0 (0)	3.67 (1.04)	4.89 (0.54)
2. care for the normal weight, healthy baby (including examination, weighing and measuring temperature)	4.83 (0.39)	5.00 (0)	3.82 (1.47)	5.00 (0)	4.37 (1.0)	5.0 (0)	4.06 (0.97)	4.90 (0.47)
3. protect the healthy baby from problems (e.g., hypothermia)	5.00 (0)	5.00 (0)	4.36 (1.21)	5.00 (0)	4.13 (1.1)	4.97 (0.3)	4.02 (1.11)	4.97 (0.18)
4. determine if a baby is properly positioned for breastfeeding	5.00 (0)	5.00 (0)	4.27 (1.27)	5.00 (0)	4.36 (1.1)	5.0 (0)	4.10 (1.07)	4.94 (0.31)
5. determine if the baby has a good attachment to the breast	4.92 (0.29)	5.00 (0)	4.55 (0.69)	5.00 (0)	4.56 (0.8)	5.0 (0)	4.00 (1.15)	4.92 (0.33)
6. ensure hygiene and provide cord care	4.92 (0.29)	5.00 (0)	3.91 (1.04)	4.91 (0.30)	4.05 (1.1)	5.0 (0)	3.92 (0.97)	4.90 (0.35)
7. give vitamin K and immunize	4.92 (0.29)	5.00 (0)	3.91 (1.45)	4.73 (0.47)	4.08 (1.1)	5.0 (0)	3.58 (1.39)	4.92 (0.33)
8. manage problems with breastfeeding	4.83 (0.39)	5.00 (0)	3.82 (1.40)	4.91 (0.30)	3.89 (1.2)	5.0 (0)	3.53 (1.11)	4.87 (0.46)
9. improve thermal care*	4.91 (0.30)	5.00 (0)	4.36 (0.92)	5.00 (0)	3.62 (1.2)	5.0 (0)	3.70 (1.10)	4.87 (0.34)
10. teach mothers to provide prolonged skin-to‐skin care	4.83 (0.39)	5.00 (0)	4.64 (0.67)	4.91 (0.30)	3.85 (1.3)	5.0 (0)	3.73 (1.19)	4.86 (0.50)
11. use alternate feeding methods	4.92 (0.30)	5.00 (0)	3.36 (1.63)	5.00 (0)	2.93 (1.5)	4.87 (0.7)	3.00 (1.32)	4.82 (0.64)
12. assess a baby for danger signs	4.83 (0.39)	5.00 (0)	4.09 (1.30)	4.91 (0.30)	3.56 (1.1)	5.0 (0)	3.81 (1.16)	4.89 (0.36)
13. give antibiotics	4.17 (0.84)	5.00 (0)	3.45 (1.44)	5.00 (0)	2.85 (1.4)	5.00 (0)	3.27 (1.32)	4.94 (0.31)
14. refer for advanced care	4.75 (0.45)	5.00 (0)	4.45 (0.82)	5.00 (0)	3.4 (1.27))	5.00 (0)	3.69 (1.22)	4.89 (0.57)
15. advise parents about care at home	4.92 (0.29)	5.00 (0)	4.09 (1.30)	5.00 (0)	4.15 (1.08)	5.00 (0)	4.00 (1.10)	4.92 (0.37)

n = 11 for pre-confidence assessment

#### Multiple Choice Question (MCQ) examination

Cognitive knowledge was assessed by administering a 28 item MCQ examination. Successful completion was defined as 80 % (22 of 28) of the total correct answers. The MCQ exam was administered to learners and facilitators in Kenya pre and post-course, and to facilitators in India post-course only. See Table 3.

**Table 3:** Multiple choice question examination of the facilitators & learners

	Facilitators		Learners			
Multiple choice question	In India (n=12) Correctly answered n (%)	In Kenya (n=11) Correctly answered n (%)	In India (n = 30)		In Kenya (n =60 )	
			Before Training	After Training	Before Training	After Training
1. How long should skin-to-skin care be provided for all babies?	7 (58.3)	8 (72.7)	3 (10.1)	17 (56.7)	16 (25.0)	54 (87.1)
2. When should babies be observed for breathing problems during the first hour after birth?	12 (100)	11 (100.0)	27 (90.0)	29 (96.7)	54 (85.7)	60 (96.8)
3. Beginning breastfeeding soon after birth is beneficial because it __________.	9 (75)	8 (72.7)	18 (60.0)	22 (73.3)	36 (57.1)	45 (73.8)
4. When should a baby be given liquids other than breast milk?	12 (100)	9 (81.8)	24 (80.0)	29 (96.7)	43 (67.2)	44 (71.0)
5. A baby who is ready to breast feed __________.	12 (100)	11 (100.0)	19 (63.3)	30 (100.0)	55 (87.3)	61 (98.4)
6. In a healthy term baby, the arms and legs __________.	11 (91.7)	11 (100.0)	22 (73.3)	26 (86.7)	55 (87.3)	59 (95.2)
7. How fast should a baby normally breathe?	12 (100)	9 (81.8)	16 (53.3)	27 (90.0)	36 (58.1)	53 (85.5)
8. What should drain from the umbilical cord one hour after birth?	10 (58.3)	10 (90.9)	21 (70.0)	26 (86.7)	36 (56.3)	51 (82.3)
9. Which is the most important reason for weighing all babies soon after birth?	12 (100)	11 (100.0)	29 (96.7)	30 (100.0)	62 (98.4)	61 (98.4)
10. Medicine to prevent eye infections should be given __________.	11 (91.7)	11 (100.0)	11 (36.7)	30 (100.0)	50 (79.4)	60 (96.8)
11. Which babies should receive eye treatment soon after birth?	3 (25)	11 (100.0)	10 (33.3)	12 (40.0)	55 (87.3)	61 (98.4)
12. Which babies should be given vitamin K after birth?	12 (100)	11 (100.0)	29 (96.7)	29 (96.7)	38 (60.3)	54 (87.1)
13. How should a baby's body temperature be maintained after skin-to-skin care?	12 (100)	11 (100.0)	28 (93.3)	30 (100.0)	63 (100.0)	62 (100.0)
14. How soon after birth should a baby first be bathed?	12 (100)	11 (100.0)	22 (73.3)	29 (96.7)	55 (88.7)	62 (100.0)
15. Good attachment for breastfeeding can be recognized when __________.	12 (100)	11 (100.0)	27 (90.0)	30 (100.0)	53 (84.1)	58 (95.1)
16. Mothers who have breast engorgement should __________.	12 (100)	11 (100.0)	23 (76.7)	30 (100.0)	58 (92.1)	59 (95.2)
17. Sore or cracked nipples may result from __________.	11 (91.7)	11 (100.0)	22 (73.3)	29 (96.7)	55 (87.3)	62 (100.0)
18. When should a second physical exam be performed if the first physical exam (during the first 90 minutes after birth) is normal?	7 (58.3)	11 (100.0)	6 (20.0)	14 (46.7)	16 (25.8)	27 (43.5)
19. What would you do if a baby has an axillary temperature of 36.0 °C two hours after birth?	12 (100)	11 (100.0)	24 (80.0)	28 (93.3)	55 (87.3)	50 (80.6)
20. When should you seek advanced care if a baby has a temperature of 36.0°C two hours after birth?	11 (91.7)	9 (81.8)	12 (40.0)	23 (76.7)	35 (60.3)	49 (79.0)
21. What should a baby wear while receiving prolonged skin-to-skin care?	12 (100)	10 (90.9)	19 (63.3)	27 (90.0)	42 (66.7)	51 (82.3)
22. A baby with which of the following conditions might benefit from being fed expressed breast milk by cup?	11 (91.7)	11 (100.0)	22 (73.3)	28 (93.3)	57 (90.5)	62 (100)
23. When cup feeding a baby, you should do which of the following?	12 (100)	11 (100.0)	18 (60.0)	26 (86.7)	35 (56.5)	56 (90.3)
24. Which of the following describes seizures?	12 (100)	11 (100.0)	24 (80.0)	29 (96.7)	47 (74.6)	53 (85.5)
25. When should a baby be given antibiotics?	12 (100)	11 (100.0)	29 (96.7)	29 (96.7)	59 (96.7)	60 (96.8)
26. When should the first dose of antibiotics be given?	12 (100)	11 (100.0)	27 (90.0)	26 (86.7)	59 (96.7)	59 (96.7)
27. What determines the dosage of a specific type of antibiotics?	12 (100)	11 (100.0)	28 (93.3)	29 (96.7)	59 (96.7)	62 (100.0)
28. Jaundice is severe when it appears on the face during the first day after birth and what other body area at any time?	12 (100)	11 (100.0)	24 (80.0)	28 (93.3)	51 (83.6)	60 (96.8)

#### Objective Structured Clinical Examinations (OSCEs)

Skills and performance were evaluated post-course only, among facilitators and learners, using two OSCEs (A and B). OSCE A evaluated the skills necessary to provide basic care during the first day after birth, and OSCE B evaluated the skills necessary for managing babies with medical problems. Participants successfully completed OSCEs if they performed 15 of 20 items correctly on OSCE A and 12 of 16 items on OSCE B. See Table 4.

**Table 4:** Knowledge, skill and performance assessment

Group	India			Kenya		
	Pre Mean (SD); Range	Post Mean (SD); Range	*P* value	Pre Mean (SD); Range	Post Mean (SD); Range	*P* value
MCQ						
• Facilitators	NA	25.8 (1.7)			26.7(0.7)	NA
		22 to 28			25 to 28	
• Learners *(30 learners in India, 62 in Kenya)*	19.4 (4.3)	24.8 (2.1)	<0.001	20.8 (3.1)	25.1 (2.9)	0.004
	6 to 26	20 to 28		11 to 27	17 to 28	
OSCE A						
• Facilitators	NA	17.6 (1.8);	NA-	NA	16.2 (1.9);	NA
		15 to 20			12 to 18	
• Learners	NA	17.6 (2.6)	NA	NA-	17.6 (2.1)	NA
		9 to 20			9 to 20	
OSCE B						
• Facilitators	NA	15.6 (1.4)	NA	NA	12.0 (1.3)	NA
		11 to 16			10 to 14	
• Learners	NA-	15.1 (1.4)	NA-	NA-	13.9 (1.6)	NA
		10 to 16			8 to 16	

NA = Not applicable

#### Qualitative evaluation and focus groups

Qualitative data were collected on evaluation forms and through discussion groups held after the educational sessions. Focus groups addressed the change in confidence, overall curriculum design, ease of completing the MCQ exam and OSCEs and the relevance of these assessments. One focus group conducted in Kenya among parents of newborns focused exclusively on acceptability and feasibility of the Parents’ Guide. The discussion groups were conducted in the local language, audio-taped, transcribed and then translated into English. One master trainer conducted the group discussion for the facilitators, and then a facilitator and/or master trainer conducted focus group discussion for learners at each of the two study sites. These focus groups used some general probes including: “would you explain further?”; “would you give me an example of what you mean?”; “would you say more?”; “was there anything else?”; “I don’t understand”. The questions raised during the discussions included feedback on specific aspects of the course, performance of each part of the course, and the potential ways to improve the course. In addition, the facilitators and learners were asked about potential obstacles which can interfere with the course dissemination and suggestions were elicited for ways to overcome these barriers. As these data focused on perceptions about the course, group discussion data were analyzed for common themes, especially for recommendations for modifications of the course material.

The study was approved by the University of Calgary, Conjoint Health Research Ethics Board and the respective sites’ ethics committees. Written informed consent was obtained from the facilitators and the learners before participation.

## Results

Twelve facilitators were trained in India and 11 in Kenya. These facilitators trained 126 learners (62 in India and 64 in Kenya). Facilitators were neonatologists, pediatricians, medical officers and nurses from level 2 or 3 neonatal care units, while learners were nurses and clinical officers from district hospitals, private nursing homes and primary health centers. The learners at both the sites had not received formal training on essential newborn care as part of their clinical orientation or ongoing work. However, the components of essential newborn care had been taught as a part of the curriculum during undergraduate training of nurses and physicians at both the sites. The majority of Indian participants (Facilitator and Learner) had no prior HBB training, In Kenya, most Facilitator (81 %) and Learner participants (86 %) were HBB-trained.

### Course assessment surveys

Facilitators in India and Kenya rated the course and their individual ability to teach the course after training as more than 4 in nearly all aspects (See Additional file 1: Table S1). Only one item ‘*I can use facilitators guide to answer questions that learner’s may ask me*’ generated a broader range of scores (range 1–5) in India but not in the Kenya study site (range 4–5). General comments about the program were mostly positive; examples include: “well planned”; “well organized.” However, some modifications for improvements in pictures and modification for local adaptation were suggested in the open-ended comments of the post-course evaluation. Facilitators also suggested possible improvements to the content of the Parent Guide and also the inclusion of more OSCEs (India). Most facilitators felt that they had sufficient ability, time and information to teach the course, and also felt that the learners would be able to use the course material and successfully complete the OSCEs and MCQ examination (See Additional file 1: Table S2). Some facilitators suggested lengthening the program schedule to three days to incorporate a supervised clinical component.

Learners felt that the course objectives were well defined, the course was well organized, sufficient time was given to learn and to practice skills and the exercises were very useful. They also felt that the learning material was explicit. They were able to understand the MCQs and would be able to perform an OSCE (Table 1).

### Confidence surveys

Among facilitators, the level of confidence in providing care was high before the course. In India, the pre-course mean score on the confidence assessment questions ranged from 4.6 to 4.9. There was a significant increase in the confidence score from pre- to post-course on only one item ‘Your confidence in how to give antibiotics’. For all items post-course scores were 5.0 and the standard deviation was zero. In Kenya, prior to the training the facilitators were quite confident in a few of the specified tasks, but their confidence ratings for seven items were below 4.0. After training, the confidence in performing tasks in seven items (#1, 2, 6, 8, 9, 11, and 13) increased (Table 2). For learners at both sites, there was a significant increase in confidence for all the tasks after completion of the course (Table 2).

### Multiple choice question examinations

Facilitators completed the MCQ examination after completion of the course only. The mean score in India was 25.8 (SD 1.7; range 22–28) and in Kenya was 26.7 (SD 0.7; range 25–28). All facilitators at both sites scored “pass” or higher (Table 3).

In India, 30 learners completed both pre- and post-course MCQ examinations. There was a significant improvement in the post-course compared to pre-course scores (mean 24.8 [SD 2.1, range 20–28] vs. 19.4 [SD 4.3, range 6 to 26]; *p* < 001). Nine learners (30 %) passed the pre-course test while 26 learners (87 %) passed the post-course test. In Kenya, 62 learners completed pre- and post-course examinations. There was a significant improvement in the post-course compared to pre-course scores (mean 25.1 [SD 2.9, range 17–28] vs. 20.8 [SD 3.1, range 11–27]; p = 0.004). Thirty learners (48 %) passed the pre-course test while 52 (84 %) passed the post-course test. The MCQ results overall suggest the course was effective in enhancing participants’ knowledge (Tables 3 and 4). However some questions were frequently answered incorrectly. In India, the items often answered incorrectly on the post-course examination included questions regarding the length of time skin-to-skin care should be provided, which babies should receive eye treatment, and the timing of a second physical examination. In Kenya, learners also often incorrectly identified the correct timing of a second physical examination.

### Objective structured clinical examinations

In India, 12 facilitators completed the OSCE A and all passed. The mean scores on the OSCE A were 17.6 (SD 1.8; range 15–20). Sixty-two learners participated in the OSCE A and 56 learners (90 %) passed. In Kenya, 11 facilitators completed the OSCE A and 9 passed with a mean score of 16.2 (SD 1.9; range 12–18). Sixty-four learners participated in the OSCE A and 60 (94 %) passed (Table 4).

In India, 12 facilitators completed OSCE B and 11 passed. Sixty-two learners completed the OSCE B and 58 (94 %) passed. In Kenya, eight of 11 facilitators passed OSCE B. Among the 59 learners who completed the OSCE B, 54 (92 %) passed. Overall learners failed to categorize the baby as this was not explicit on the Action Plan.

### Qualitative evaluation and focus groups

**Fig. 2:**
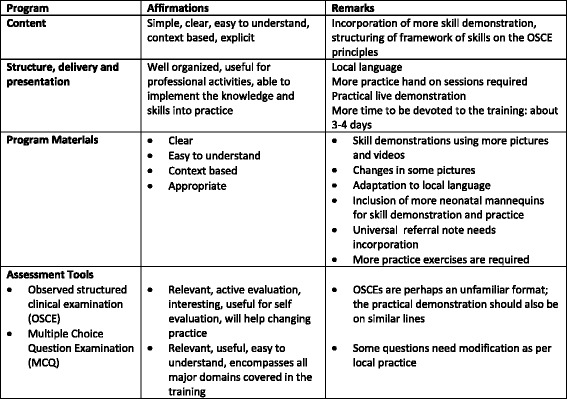
Main points from the focus group discussion with facilitators and the course evaluation

In India, a total of 3 focus groups (facilitators = 1, learners = 2) were conducted, and in Kenya, a total of 4 (facilitator = 1; learners = 2; parent’s guide = 1). Regarding the structure of the course, some learners suggested that more time should be devoted to teaching and practicing. Participants also suggested that videos and or pictures would add more value to the current structure of learning. In addition, they recommended changing some MCQ questions, making them more explicit or changing the options available. All learners felt that the material was simple, understandable, and easy to learn and teach. Some learners expressed a desire for more practical hands-on sessions, a need for more neonatal simulators and the need for practice sessions in the clinical setting. Participants spoke about the importance of training both physicians and nurses in their workplace and aligning procurement of supplies and equipment with the recommended practices. They identified some potential changes needed in policies and procedures in order to translate the knowledge and skills gained into practice. The facilitators suggested that a few modifications in the content would be necessary so that the content would be consistent with approved local practices (for example an approved alternative guideline for eye care). They also suggested some pictorial modifications in the flip chart and more structured clinical examinations, rewording of some lines in the OSCEs and a universally adaptable referral note. Facilitators, Learners, and parents of newborns 6 weeks or younger enthusiastically embraced the Parent’s Guide, particularly the fact that it is colorful, graphically based, and reinforces key messages related to preservation of newborn health after discharge, including exclusive breast feeding, hygiene, and recognition of danger signs. Participants suggested that the Parent’s Guide might serve as a useful linkage between facility and community-based efforts to educate family and newborn care providers. The main points from the focus group discussion and course evaluation are provided in Fig. 2.

### Information from observations of facilitator teaching

In addition to the formal evaluations described above, in both India and Kenya master trainers observed the program being taught by the newly trained facilitators. This observation allowed the master trainers to identify gaps in teaching and potential areas of improvement in teaching materials. The master trainers learned that it was difficult for learners to categorize babies after the first 90 min following birth, a required step in determining subsequent care. As a result of this observation, a page outlining this critical step was added to the Facilitator flip chart and Provider Guide.

## Discussion

The ECEB program is a simplified educational program designed to facilitate the implementation of evidenced-based newborn care practices in resource-limited environments. In this manuscript, we report the results of a formative evaluation of the program at two international sites. At each site, we collected data during the training of new facilitators and during their subsequent training of community-level providers (called learners in this study). Both facilitators and providers (learners) felt that the structure and content of the program were appropriate for their environments and suitable for teaching and learning. The program increased knowledge among providers, and nearly all facilitators and providers acquired sufficient skills necessary to perform essential newborn care. Given the simplicity of the program and its apparent suitability for a train-the-trainer strategy of dissemination, it may be an ideal program for increasing knowledge and skills of providers in essential newborn care in resource-limited environments.

Confidence in performing newborn care practices was assessed by the administration of a survey. In India, before completing the course, facilitators were quite confident about their ability to perform nearly all tasks, while in Kenya, pre-course confidence among facilitators was somewhat lower. All facilitators in India and nearly all in Kenya indicated they were fully confident in their ability to perform all tasks after completing the program. Because providers who participated in this study were, in general, less educated and experienced than facilitators, their baseline confidence in performing newborn care was lower than facilitators’ confidence. However, among providers in both countries, increases in confidence in performing all aspects of essential newborn care were observed.

In both India and Kenya, provider knowledge, assessed by the MCQ examination, improved significantly from pre-course to post-course. In Kenya, Facilitator knowledge also improved. These results suggest that overall the program was effective in helping participants acquire the intended knowledge. However, a few items were not answered correctly by a relatively large number of providers. This suggests that these particular topic areas may not have been adequately addressed in the training. An alternative explanation is that some practices may be misunderstood such as provision of Vitamin K in the Kenya. Learner feedback suggested that due to a combination of misunderstanding the relative risk of all newborns developing bleeding disorders in the absence of vitamin K injection; and frequent insufficient supplies of vitamin K, some learners held the erroneous assumption that only premature infants should routinely receive a vitamin K injection immediately after birth. It is also possible that specific country guidelines that may differ from the program content led to confusion, and the providers answered the questions based on the country guidelines and their usual practice. This may suggest the need to modify the program content to conform to evidence-based guidelines recommended by local health authorities (e.g. eye or cord care). Some providers may have had insufficient time to complete the examination due to limited language skills or literacy, or lack of familiarity with the MCQ format. Finally, we did not evaluate long-term retention of knowledge in this study. As has been observed with other training programs, it seems likely that some decrement in knowledge and skills will occur over time. In the Provider Guide, strategies for maintaining knowledge and skills are recommended.

The results of the performances on the OSCEs suggest that completion of the ECEB course results in the acquisition of the skills necessary to provide essential newborn care among nearly all learners. Since the acquisition of skills was similar among facilitators and providers, the results suggest that the ECEB course is effective at teaching skills to a diverse group of health care professionals with varied clinical experience. However, up to 10 % of learners failed to achieve a passing score on one of the OSCESs. A passing score was intended to identify the skills necessary to deliver adequate essential newborn care. It is possible that some learners may need additional training to acquire these skills. It is also possible that the OSCEs are not a true measure of clinical skills. A few facilitators also failed to achieve a passing score, which could be due to inadequacies in OSCE’s. Following this beta testing, some modifications were made in the OSCEs. We plan to evaluate the impact of these changes.

Opinions regarding potential areas for improvement of the program were also gathered during focus group discussions. This information suggested the need for some amendments in the program. One theme that emerged was the need to tailor the time devoted to the program to the background and needs of the learners. The course was designed to be conducted during 12 h of classroom instruction. Some participants expressed a need for more time for teaching and hands-on practice in the actual clinical setting. The ideal duration of the program may be variable and depend upon baseline levels of training and clinical expertise among learners. It appears that the program is acceptable, feasible, and effective at increasing knowledge and skills in essential newborn care among participants who are both HBB-trained and HBB-naïve. Some suggested the potential benefit of supplementing the classroom teaching with training in a clinical setting.

The ECEB program is intended to establish adequate and uniform knowledge and skills among providers of newborn care. This an essential step in implementing newborn care practices. However, translation of knowledge of skills into practice will also require overcoming barriers to implementation, which might include inadequate commodities, ill-suited facilities and cultural barriers to adoption and change. We believe that the ECEB program will result in change in practices and outcomes only if it is incorporated into an implementation plan that includes monitoring and evaluation and quality improvement strategies to overcome barriers to implementation.

There are several limitations to this study. Prior exposure to the teaching strategy used in ECEB varied between sites. In Kenya, facilitators had all been trained using the HBB program; in India, care at the time of birth had been taught using a similar but not identical program NSSK (Navjat Shishu Surkshya Karyakram). This difference may have influenced the relative change in acquisition of skills and knowledge between sites. Change in knowledge was not tested among all learners. A pre-training MCQ examination was not administered to facilitators because we assumed that they all had a high knowledge base. The pre-training MCQ examination was only administered to providers in one of the two Indian sites because of practical problems. We chose not to administer the OSCEs prior to training because performance on the OSCEs is somewhat dependent upon the sequence of care that is introduced in the ECEB program. Therefore, we did not believe that the OSCEs were a fair assessment of baseline skills. The OSCEs were used solely to test the assimilation of knowledge and adequacy of skills at the completion of training. Finally, we did not evaluate long-term retention of knowledge and skills, nor did we evaluate whether the knowledge and skills acquired during training would be translated into improved care.

### Future directions

We hope that the ECEB program will be a vital resource for improving the health of newborns in low-resource environments. At the time of this writing, the program has been introduced to representatives from countries in Africa and South East Asia. These individuals were informed about the educational strategy and content of the program and advised about the need to adapt the program to be compliant with the policies for newborn care of their respective health authorities. We presume that their decisions about whether to use the program will be based on how the ECEB program might interface with or replace ongoing educational programs. We also recognize that the use of the ECEB program needs further investigation. Acquisition of knowledge and skills is an enabling step in improving care, but this step must be incorporated into a broader implementation strategy to effect change. To understand the potential impact of ECEB training, its effectiveness in changing processes and clinical outcomes must be tested in future studies. These studies should test effectiveness in the context of the accompanying elements of implementation.

## Conclusion

Our evaluation of the ECEB program, a simplified program for teaching essential newborn care, suggests that the program has several attributes of an ideal program to support implementation and adaptation of these practices in resource-limited environments. The program was judged by facilitators (teachers) to be easy to use and suitable for teaching in their environments. Learners (community-based providers) nearly all demonstrated adequate skills after training. The program appears to be appropriate for dissemination using a train-the-trainer strategy. Participants also highlighted the importance of additional practice and the need for changes in organizational systems in order to achieve improved patient outcomes. Successful translation into improved care will require strengthening of health care systems.

## Additional file

Additional file 1: Table S1. Facilitators’ Post-Course Evaluation. **Table S2.** Facilitators’ Post-Teaching Course Evaluation.

## Abbreviations

AAP: American Academy of Pediatrics
ECEB: Essential Care for Every Baby
HBB: Helping Babies Breathe
MCQ: Multiple Choice Questions
OSCE: Objective Structure Clinical Evaluation
WHO: World Health Organization
