# Supplementary material for: Evaluation of an educational program for essential newborn care in resource-limited settings: Essential Care for Every Baby
Source: BMC Pediatr. 2015 Jun 24;15:71. doi: 10.1186/s12887-015-0382-z (PMC4479066; doi:10.1186/s12887-015-0382-z)
Supplement: Additional file 1: Table S1. — Facilitators’ Post-Course Evaluation. Table S2. Facilitators’ Post-Teaching Course Evaluation. [file 12887_2015_382_MOESM1_ESM.docx]

**Additional File Table 1: Facilitators’ Post-Course Evaluation**

|  | India  (n=12)  Mean (SD) | Kenya  (n=11)  Mean (SD) |
| --- | --- | --- |
| 1. The course objectives are clear | 4.75 (0.45) | 5.00 (0) |
| 2. The course is well-organized | 4.92 (0.29) | 4.91 (0.30) |
| 3. The course is designed in a systematic way | 4.92 (0.29) | 4.91 (0.30) |
| 4. I had sufficient time to learn how to teach the course | 5.00 (0) | 4.55 (0.69) |
| 5. Course materials were easy to read | 5.00 (0) | 4.82 (0.40) |
| 6. Course materials were easy to understand | 5.00 (0) | 4.90 (0.32) |
| 7. I know what is expected of me to teach the course | 4.92 (0.29) | 4.55 (0.52) |
| 8. I have enough information to teach the course | 4.83 (0.39) | 4.55 (0.52) |
| 9. I have the skills to teach the course | 4.75 (0.45) | 4.73 (0.47) |
| 10. I will be able to use the course objectives to guide my teaching | 4.92 (0.29) | 5.00 (0) |
| 11. I can use the Facilitator Guide to answer questions that learners may ask me | 4.27(1.3) | 4.91 (0.30) |
| 12. I can teach learners how to use the Provider Guide | 4.83 (0.39) | 4.91 (0.30) |
| 13. There are enough visual materials to understand the key concepts | 4.67 (0.49) | 4.55 (0.93) |
| 14. I can use the exercises to help learners consolidate information | 4.67 (0.49) | 4.73 (0.47) |
| 15. I can teach the learners the information they need to succeed at the multiple choice examination | 4.67 (0.49) | 4.64 (0.50) |
| 16. I can teach my learners how to successfully perform the combined skills in the OSCEs | 5.00 (0) | 4.82 (0.40) |
| 17. I can run an OSCE | 5.00 (0) | 4.64 (0.50) |
| 18a. My learners will be able to use the Action Plan | 4.75 (0.45) | 4.73 (0.47) |
| 18b. My learners will be able to use the Provider Guide | 4.83 (0.39) | 4.73 (0.47) |
| 18c. My learners will be able to use the Parent Guide | 4.67(0.65) | 4.82 (0.40) |
| 19a. My learners will be able to complete the "Facilitate practice" exercises | 4.50 (0.52) | 4.73 (0.47) |
| 19b. My learners will be able to complete the OSCEs | 4.42(0.52) | 4.73 (0.47) |
| 19c. My learners will be able to complete the Multiple-choice question (MCQ) exam | 4.58 (0.52) | 4.73 (0.47) |
| 20. I am looking forward to teaching this course | 4.83(0.39) | 4.91 (0.30) |

**Additional File Table 2: Facilitators’ Post-Teaching Course Evaluation**

|  | **India** (n=10)  Mean (SD) | **Kenya** (n=8)  Mean (SD) |
| --- | --- | --- |
| 1. I was able to teach the course as planned | 4.80 (0.42) | 5.0(0) |
| 2. I had sufficient time to teach the course | 4.80 (0.42) | 4.63(0.73) |
| 3. I had enough information to teach the course | 4.90 (0.32) | 4.88(0.35) |
| 4. I had the skill to teach the course | 4.90 (0.32) | 5.0(0) |
| 5. I could answer the questions that the learners asked | 5.00 (0) | 5.0(0) |
| 6. I taught the learners the information they need to succeed at the multiple choice examination | 4.70 (0.48) | 4.88(0.35 |
| 7. I taught the learners how to successfully perform the combined skills in the OSCEs | 5.00 (0) | 5.0(0) |
| 8. The learners were able to learn how to complete an OSCE | 5.00 (0) | 5.0(0) |
| 9. The learners were able to understand the content of the Provider Guide | 4.90 (0.32) | 4.88(0.38) |
| 10. I was able to teach the learners how to use the Provider Guide | 4.90 (0.32) | 5.0(0) |
| 11. I was able to use the exercises to help the learners consolidate information | 4.70 (0.95) | 5.0(0) |
| 12a. The learners will be able to use the Action Plan | 5.00 (0) | 5.0(0) |
| 12b. The learners will be able to use the Facilitator's Flip Chart | 4.90 (0.32) | 4.88(0.35) |
| 12c. The learners will be able to use the Provider Guide | 4.90 (0.32) | 4.88(0.35) |
| 12d. The learners will be able to use the Parent Guide | 4.89 (0.33) | 4.63 (0.74) |
| 13a. The learners will be able to complete the "Facilitate practice" exercises | 4.80 (0.42) | 4.88(0.35) |
| 13b. The learners will be able to complete the OSCEs | 4.89 (0.33) | 5.0(0) |
| 13c. The learners will be able to complete the Multiple-choice question (MCQ) exam | 4.80 (0.42) | 5.0(0) |
| 14. I am looking forward to teaching this course | 4.90 (0.32) | 5.0(0) |
| 16. Learners found the Action Plan useful | 5.00 (0) | 4.86(0.38) |
| 17. Learners found the multiple choice question examination acceptable | 4.70 (0.48) | 4.71(0.49) |
| 18. Learners understood the questions asked on the multiple choice examination | 4.80(0.42) | 4.57(0.53 |
| 19a. maintain skin-to-skin care and when to separate the baby and mother | 4.90 (0.32) | 4.71(0.49) |
| 19b. care for the normal weight, healthy baby (inc. examination, weighing and measuring temp.) | 4.90 (0.32) | 4.86(0.38) |
| 19c. protect the healthy baby from problems (e.g.hypothermia) | 4.80(0.42) | 5.0(0) |
| 19d. determine if a baby is properly positioned for breastfeeding | 4.90 (0.32) | 5.0(0) |
| 19e. determine if the baby has a good attachment to the breast | 4.90 (0.32) | 4.71(0.49) |
| 19f. ensure hygiene and provide cord care | 5.00 (0) | 4.86(0.38) |
| 19g. give vitamin K and immunize | 5.00 (0) | 4.57(0.79) |
| 19h. manage problems with breastfeeding | 4.80 (0.42) | 4.71(0.49) |
| 19i. improve thermal care | 4.90 (0.32) | 5.0(0) |
| 19j. teach mothers to provide prolonged skin-to-skin care | 5.00 (0) | 4.86(0.38) |
| 19k. use alternate feeding methods | 4.60 (0.70) | 4.86(0.38) |
| 19l. assess a baby for danger signs | 4.70 (0.48) | 4.57(0.79) |
| 19m. give antibiotics | 4.90 (0.32) | 4.57(0.79) |
| 19n. refer for advanced care | 4.80 (0.42) | 5.0(0) |
| 19o. advise parents about care at home | 4.90 (0.32) | 4.71(0.49) |
